# QTL Mapping and Validation of Adult Plant Resistance to Stripe Rust in Chinese Wheat Landrace Humai 15

**DOI:** 10.3389/fpls.2018.00968

**Published:** 2018-07-05

**Authors:** Feng-Ping Yuan, Qing-Dong Zeng, Jian-Hui Wu, Qi-Lin Wang, Zu-Jun Yang, Bang-Ping Liang, Zhen-Sheng Kang, Xin-Hong Chen, De-Jun Han

**Affiliations:** ^1^College of Agronomy, Northwest A&F University, Yangling, China; ^2^Shaanxi Key Laboratory of Genetic Engineering for Plant Breeding, Yangling, China; ^3^State Key Laboratory of Crop Stress Biology for Arid Areas, Northwest A&F University, Yangling, China; ^4^College of Plant Protection, Northwest A&F University, Yangling, China; ^5^School of Life Science and Technology, University of Electronic Science and Technology of China, Chengdu, China

**Keywords:** adult plant resistance (APR), fluorescence *in situ* hybridization (FISH), *Puccinia striiformis* f. sp. *tritici*, quantitative trait loci (QTL), *Triticum aestivum*

## Abstract

Stripe rust caused by *Puccinia striiformis* f. sp. *tritici* (*Pst*) is a devastating foliar disease that affects common wheat and barley throughout the world. The reasonable deployment of adult plant resistance (APR) wheat varieties is one of the best methods for controlling this disease. Wheat landraces are valuable resources for identifying the genes/QTLs responsible for disease resistance. Humai 15 is a Chinese spring wheat landrace and it has exhibited adequate levels of APR to the prevalent *Pst* races in field environments for many years. In this study, a population of 177 recombinant inbred lines (RILs) was derived from Humai 15 × Mingxian 169. After screening based on a 90K chip array using 45 RILs and Kompetitive Allelic Specific PCR marker genotyping for the population of RILs, a major effect QTL in Humai 15 was located on the centromere of chromosome 2B, where it accounted for up to 47.2% of the phenotypic variation. Two other minor QTL genes from Humai 15 were located on chromosome arms 3BS and 4BL. The *Yr18* gene was identified on chromosome arm 7DS in Mingxian 169.

## Introduction

Stripe (yellow) rust caused by *Puccinia striiformis* Westend. f. sp. *tritici* Erikss. (*Pst*) is one of the most important foliar diseases of the winter wheat throughout the world. Stripe rust epidemics occur mainly in the winter wheat regions in northwestern, southwestern, and northern China, and the major spring wheat areas in northwestern China (Li and Zeng, 2002; Wan et al., 2004; Chen et al., 2009), and the northwest and southwest regions are the important over-summering areas, e. g., Shaanxi, Gansu, and Sichuan provinces. This disease badly affects wheat production and can cause crop losses of up to 100%, although the losses usually range from 10 to 70% (Chen, 2005). Growing resistant cultivars is the most effective, economical, and environmentally friendly approach for controlling stripe rust (Röbbelen and Sharp, 1978; Line and Chen, 1995).

Resistance to stripe rust can be classified into all stage resistance (ASR) and adult plant resistance (APR) according to the growth stage. All stage resistance, also called seedling resistance, is race specific and inherited qualitatively as monogenic resistance, and thus it is usually not durable. APR plants are susceptible at the seeding stage and resistant in later stages. APR is controlled by multiple genes and the performance can be classified as quantitative resistance. Plants with all stage resistance are highly resistant and readily handled by the breeder, and many ASR *Yr* genes are used in numerous elite varieties, although they are prone to becoming susceptible when new pathogen races emerge. By contrast, pyramiding multiple QTLs into elite varieties can provide durable resistance to stripe rust, such as “Rely.” The phenotypes are difficult to assess for plants with APR genes and this can be a time- consuming process depending on the environment and adult stage, but molecular markers linked with QTLs can accelerate breeding program.

At present, 80 *Yr* resistance genes have been formally catalogued (https://shigen.nig.ac.jp/wheat/komugi/genes/symbolClassList.jsp), but APR genes comprise a minority, where several gene loci are pleiotropic for biotrophic fungal diseases, such as *Yr18*/*Lr34*/*Pm38*/*Sr57, Yr29*/*Lr46*/*Pm39*/*Sr58, Yr30*/*Lr27*/*Pm48*/*Sr2*, and *Yr46*/*Lr67*/*Pm46*/*Sr55*. During the last 18 years, more than 160 QTL have been tentatively designated in 49 regions of 21 chromosomes (Rosewarne et al., 2013; Maccaferri et al., 2015). Several regions contain more than one gene but further delineation of these regions has not been possible due to the limitations of consensus mapping and sequentially highlighting the most important regions in wheat breeding programs (Rosewarne et al., 2013). A QTL gene usually has small effects on reducing the severity of disease, but the presence of several QTLs or their combination with an effective all stage resistance gene can be comparable to immunity and provide more durable resistance (Singh et al., 2000; Agenbag et al., 2014).

Most QTLs do not have effective markers for marker-assisted selection due to the limitations of high-density mapping and markers. The ability of simple sequence repeat (SSR) markers to saturate the wheat genome is limited (Somers et al., 2004). Since the release of the draft sequence of hexaploid bread wheat, the development and application of genome sequencing and single nucleotide polymorphisms (SNPs) in wheat has overcome various obstacles, and genome-wide association mapping has also proved a promising tool for capturing trait loci and characterizing the effects of those loci (Mayer et al., 2014; Zegeye et al., 2014; Yang et al., 2015; Liu et al., 2017). SNP arrays and Kompetitive allele-specific PCR (KASP) based on SNP genotyping have accelerated the efficiency and throughput of marker screening in order to obtain high-density genetic maps.

Humai 15 is a spring wheat variety from Qinghai province, which was registered in 2006 but there is no information about its genealogy (Li et al., 2012; Chen et al., 2013). Tests at the seedling and adult plant stages conducted in Shaanxi and Gansu provinces have shown that Humai 15 is highly susceptible to the prevalent *Pst* races CYR32, CYR33, and PST-V26 at the seedling stage, but it has a high level of resistance to stripe rust in the field in China (Han et al., 2012). Previously, four linkage maps were constructed using SSR molecular markers by mapping a population of the F_2:3_ family derived from the cross of Mingxian 169 and Humai 15 (unpublished data). In the present study, we improved the resolution of the QTL on chromosome 2B by developing a population of recombinant inbred lines (RILs) from the F_2:3_. The objectives of this study were: (i) fine mapping of the QTL for stripe rust resistance using 90K and 660K SNP arrays, (ii) identifying candidate resistance genes in the target region, and (iii) developing robust KASP-SNP markers for marker-assisted selection.

## Materials and methods

### Plant materials

In the present study, two mapping populations were developed from the cross between Humai 15 and Mingxian 169 comprising 181 F_2:3_ and 177 F_5:7_ (represent F_5:6_ and F_6:7_) RILs. The RILs were generated by single seed descent. Humai 15 is a type of spring wheat that is highly resistant to Chinese *Pst* races at the adult plant stage, and it was provided by Prof. Wanquan Ji, Northwest A&F University, China. Mingxian 169 is a Chinese winter wheat landrace that is highly susceptible to the currently prevalent *Pst* races at all stages in China. Mingxian 169 and Xiaoyan 22 were used as susceptible control varieties. We used 149 wheat varieties collected from all over the world for haplotype analysis.

### Field trials

To identification the stable QTLs, field tests of the F_5:6_ and F_6:7_ RILs were conducted in Yangling (Shaanxi Province), Tianshui (Gansu Province, over-summering region for stripe rust in China, about 300 km from Yangling), and Jiangyou (Sichuan Province, over-wintering region for stripe rust in China, about 560 km from Tianshui and 600 km from Yangling) during the 2015–2016 and 2016–2017 cropping seasons, respectively. Limited by the seeds only two biological replicates were performed in each place. The F_2:3_ lines were tested in Yangling and Tianshui during the 2012–2013 season. In each year, each location was considered to be a single environment. For the F_2:3_, we conducted two randomized replicates in Yangling and one site in Tianshui with the limitation of the F_2_ seeds. The field data obtained for the RILs and F_2:3_ comprised data for six environments and two environments, respectively. At each location, we prepared a row with a length of 1 m and a distance of 0.3 m between the rows. Approximately 30 seeds from the parents or offspring lines were sown in each row. The wheat cultivar Xiaoyan 22 was planted every 20 rows to aid the spread of the pathogen during the trial. To ensure that the abundance of the field inoculum was sufficient, Mingxian 169 was sown in spreader rows and it was also planted perpendicularly around the field. The cultural practices commonly used for wheat production in terms of fertilization and irrigation in the test regions were used to manage the nurseries in Tianshui and Jiangyou, and artificial inoculation was not performed. In the Yangling nursery, the plants were inoculated with a mixture of the prevalent Chinese *Pst* races comprising CYR32, CYR33, and PST-V26. The populations in all environments were scored for adult plant responses based on the infection types (IT) and disease severity (DS). IT and DS scores were recorded three times for each line, when the stripe rust severity in Mingxian 169 reached approximately 40, 60, and 95% during April 1–15 in Jiangyou, May 1–20 in Yangling, and May 25 to June 15 in Tianshui, respectively. IT was recorded based on a scale of 1–9 according to Line and Qayoum (1992). DS was scored as the percentage of the diseased leaf area. Maximum disease severity (MDS) scores were also used in the genetic analyses. The area under the disease progress curve (AUDPC) was computed according to the method described by Shaner and Finney (1977) and converted into the relative AUDPC (rAUDPC).

### SNP arrays analysis

Plants from the parents and progeny lines were grown in a greenhouse up to the two-leaf stage without light in order to obtain etiolated leaf tissues for the isolation of genomic DNA. Genomic DNA was extracted from the leaves using the CTAB method, as described by Murray and Thompson (1980). In order to detect the QTLs related to stripe rust resistance, the polymorphic SSR markers in the parents and bulk samples were genotyped in the F_2:3_ population. In the population of RILs, 45 F_5:6_ lines were randomly selected as well as their parents and subjected to genome-wide scanning using an Illumina wheat 90K iSelect SNP array. In addition, resistant and susceptible bulk samples were prepared with equal amounts of DNA from seven highly resistant and seven highly susceptible F_6:7_ plants. The parents, resistant DNA bulk samples, and susceptible DNA bulk samples were genotyped with an Affymetrix wheat 660K array. All of the genotyping experiments were performed by CapitalBio Corporation (Beijing, China). SNP genotype calling and clustering were performed as described by Wu et al. (2018). The flanking sequences of all the SNP probes were subjected to BLAST against the Chinese Spring reference sequence (version 1.0 http://202.194.139.32/blast/viroblast.php) in order to determine their physical positions.

### Marker assays

The two parents were also screened using 1192 SSR primers, where SSR markers with polymorphisms were used to genotype the F_2:3_ genetic populations. The SSR assays were implemented as described by Wu et al. (2018). Based on the chromosomal locations of polymorphic SNPs, KASP markers were developed for polymorphic SNPs in 90K and 660K assays via the software PolyMarker (Ramirez-Gonzalez et al., 2015a). KASP and SSR markers corresponding to the chromosome were used to screen between the parents and contrasting DNA bulk samples. The SSR and KASP PCR reactions mixtures and program cycles were as described by Wu et al. (2018).

### Statistical analysis

The rAUDPC for F_2:3_ and the MDS for each RIL in each environment were subjected to analysis of variance (ANOVA). ANOVA and Pearson's correlation coefficients were calculated using QTL IciMapping V4.1 (Wang, 2009; Meng et al., 2015). The variance was used to calculate the broad sense heritability (*h*_*b*_^2^) of stripe rust resistance: *h*_*b*_^2^ = ${\sigma \;}_{g}^{2}$/ (${\sigma \;}_{g}^{2}$ + ${\sigma \;}_{ge}^{2}$/e + ${\sigma \;}_{\varepsilon }^{2}$/*re*), where $\sigma_{g}^{2}$, $\sigma_{ge}^{2}$, and $\sigma_{\varepsilon }^{2}$ are estimates of the genotype, genotype × environment interaction, and error variances, respectively, *r* = number of replicates, and *e* = number of environments.

### Genetic map construction and QTL analysis

Genotypic SNP markers were first binned using the BIN function in QTL IciMapping V4.1. The filtered markers were then used to construct linkage maps with the MAP function in QTL IciMapping V4.1 and maps were drawn using Mapchart V2.3 (Voorrips, 2002). Logarithm of odds (LOD) score thresholds ranging from 5 to 8 were set to divide the linkage groups.

Genotypic data for the SSR and KASP markers were used to construct genetic linkage maps with QTL IciMapping V4.1. The map distance (centimorgans, cM) was converted by recombination fractions using the Kosambi function. The QTL locations and effects were analyzed with QTL IciMapping V4.1 by inclusive composite interval mapping (ICIM) analysis. The threshold LOD score was set at 2.5 (manual input) to detect significant QTLs. The phenotypic variances explained (PVE) by individual QTLs were also obtained using ICIM.

### Information and expression data for genes located within the 2B physical intervals

Annotated genes within the physical intervals were extracted from the iwgsc_refseqv1.0 gene annotation file. In order to obtain homoeologs of the annotated genes in TGAC (Clavijo et al., 2017), we employed the same approach described by Pfeifer et al. (2014). We downloaded the transcript per million (TPM) gene expression values for previously mapped RNA-seq samples from www.wheat-expression.com (Borrill et al., 2016). The transformed TPM value log^2^ (TPM+1) were visualized using the heatmap.2 function in the gplots package in R v3.3.3.

### Haplotype analysis of the major QTL located on 2B

The probes located within physical intervals on Chinese Spring chromosome 2B were used in comparisons based on the genetic and physical distances. The genotype data were retrieved for these probes in 149 wheat lines and aligned. The dendextend package in R (v3.2.2) was used for hierarchical clustering based on genotypic and phenotypic kinship. Data were visualized using the Heatmap function in the ComplexHeatmap package in R.

### Fluorescence *in situ* hybridization (FISH) analysis of the parent cultivars

The probes Oligo-pSc119.2-1 and Oligo-pTa535-1 were used for FISH analysis of wheat cultivars Humai 15 and Mingxian 169 to detect structural variations in the wheat chromosomes. Oligo-pSc119.2-1 was 5′ end-labeled with 6-carboxyfluorescein (6-FAM, *green*) and Oligo-pTa535-1 was 5′ end-labeled with 6-carboxytetramethylrhodamine (Tamra, *red*). The sequences and the process used for preparing the probes were described by Tang et al. (2014). Probe labeling, *in situ* hybridization, and chromosome spreads of materials were performed as described by Han et al. (2006).

## Results

### Phenotypic evaluation

**Figure 1:**
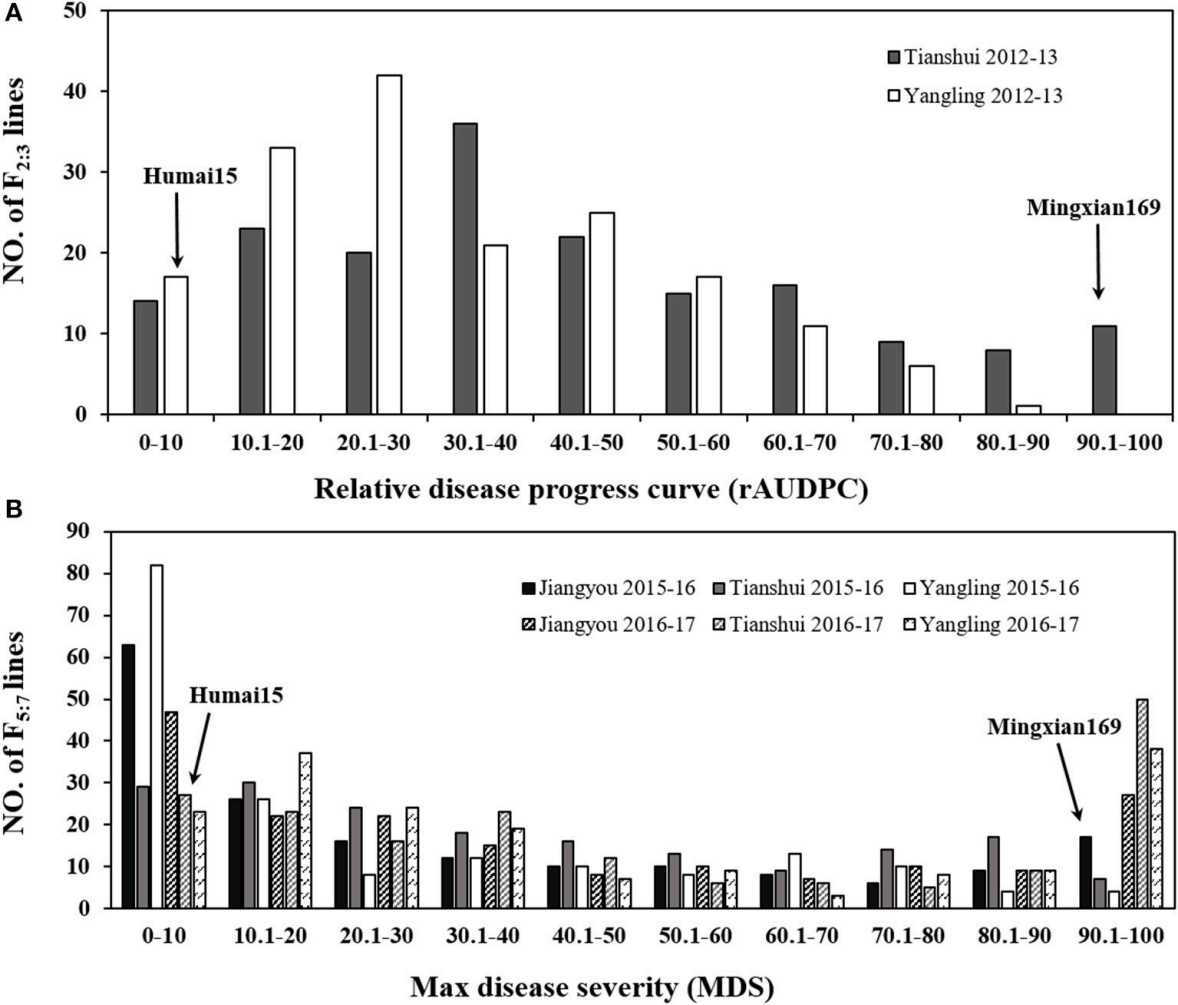
Frequency distribution of the relative area under the disease progress curve (rAUDPC) for the F_2:3_ population in Yangling and Tianshui during 2012–2013 **(A)**. Maximum disease severity (MDS) for the 177 RILs from Mingxian 169 × Humai 15 evaluated in Yangling, Jiangyou, and Tianshui during 2015–2016 and 2016–2017 **(B)**. Mean values for the parents, Mingxian 169, and Humai 15 are indicated by arrows.

In all of the environments for the field tests, Humai 15 had scores of IT 0–2 and DS = 0–10, with a mean MDS = 2.08, whereas Mingxian 169 had scores of IT = 8–9 and DS = 80–100, with a mean MDS = 87.5. The average rAUDPC for the F_2:3_ lines was 37.6%, where the value ranged from 32.1 to 43.1% in Yangling and Tianshui during 2012–2013. The average MDS for the 177 RILs was 40.52%, where it ranged from 26.7 to 52.7% in all of the environments. The frequency distributions of the mean IT and mean MDS for the 177 RILs lines as well as the mean rAUDPC for the F_2:3_ lines exhibited continuous variation in all of the environments (Figure 1), thereby indicating polygenic variation. The MDS data were significantly correlated for the population of RILs (*P* < 0.001, 0.73–0.94) in the six environments (Table S1), and the broad sense heritability was 0.95 across all environments (Table 1). ANOVA detected significant phenotypic variation in MDS among the RIL lines as well as the rAUDPC for the F_2:3_ lines, environments, and line × environment interactions in the field experiments (Table 1). These results suggest that genes had large effects on the interaction between wheat and *Pst* races in different environments.

**Table 1:** Analysis ofvariance of rAUDPC scores for F_2:3_ and MDS for F_5:7_ lines generated from Humai 15/Mingxian 169.

**Source**	**rAUDPC of F**_**2**:**3**_			**MDS of F**_**5**:**7**_		
	**Df**	**Mean square**	***F*-value**	**Df**	**Mean square**	***F*-value**
line	173	1,662	19.1^*^	176	11,479	2,398^*^
Environment	1	11,959	137.5^*^	5	279,999	584^*^
line*Environment	172	159	1.8^*^	880	253	5^*^
Error	345	87		1,056	48	
*${\text{h}}_{b}^{2}$*	0.904			0.945		

* *Significant at P < 0.001*.

${\text{h}}_{\text{b}}^{2}$, broad-sense heritability.

### QTL analysis of the F_2:3_ population

**Figure 2:**
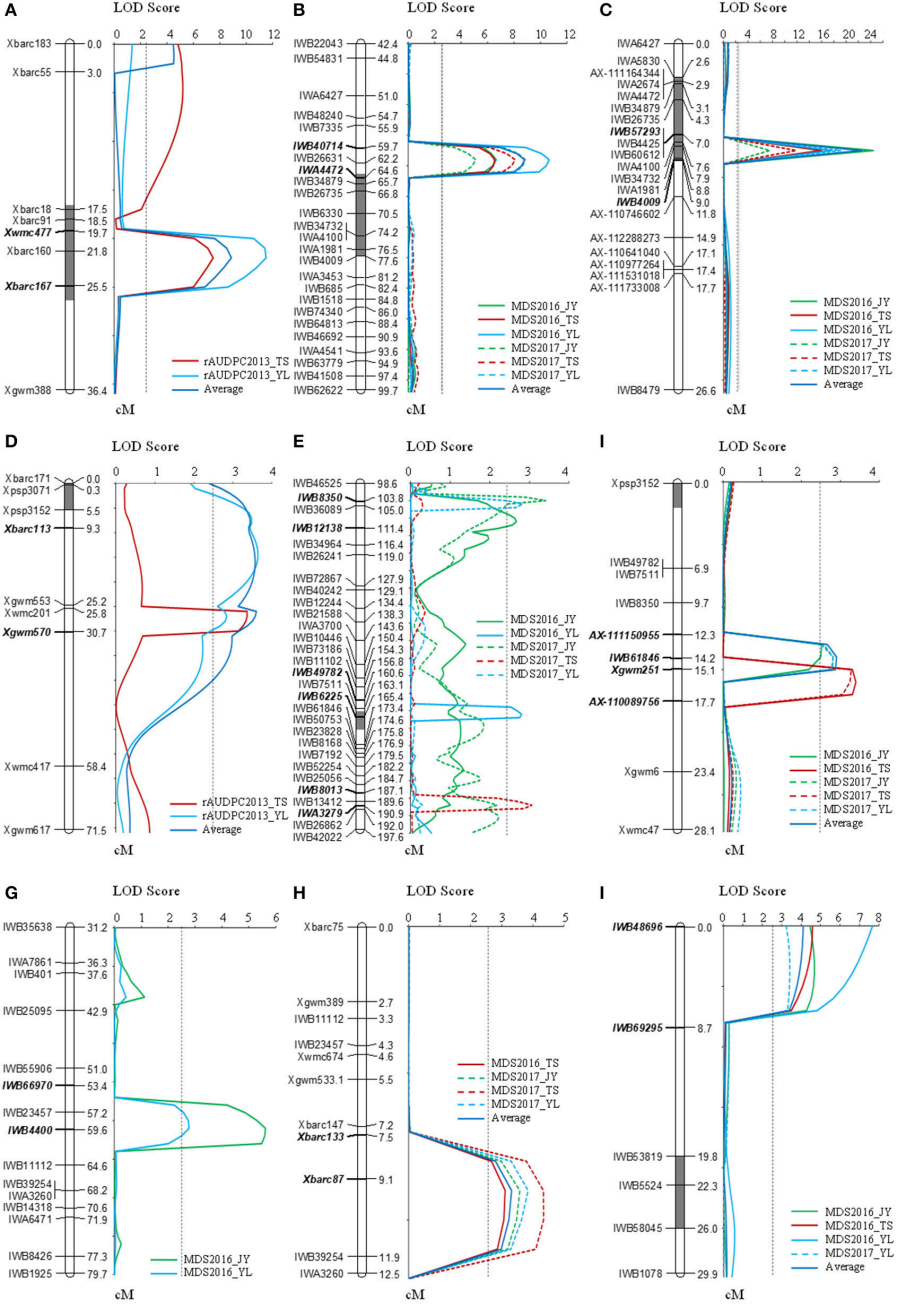
QTLs related to stripe rust resistance on chromosomes 2B **(A–C)**, 4B/6A **(D–F)**, 3B **(G,H)**, and 7D **(I)**. The QTLs were detected based on the relative area under the disease progress curve (rAUDPC) in the F_2:3_ population **(A,D)**, and by MDS in sub-populations **(B,E,G,I)** and the population of 177 RILs **(C,F,H)**. The QTLs were identified by inclusive composite interval mapping (ICIM) with LOD peak values of more than 2.5.

The F_2:3_ population of the Mingxian 169 and Humai 15 cross was used for detecting and mapping stripe rust QTLs. We found that 43 SSR markers exhibited polymorphism between the parents and bulk samples, where we constructed four linkage maps based on chromosomes 2B, 3B, 6A, and 5B/7B. QTL ICIM analysis detected two QTLs on chromosomes 2B and 6A (Table 2, Figure 2). The QTL on chromosome 2B was stable and the major QTL with a PVE of 19.7–26.2% in the marker interval of *Xbarc160* and *Xbarc167*. The QTL on chromosome 6A was a minor QTL and it explained 8.1–8.4% of the phenotypic variation.

**Table 2:** Summary of stripe rust resistance QTL in each given year using the area under the disease progress curve (rAUDPC) for F_2:3_ and the maximum disease severity (MDS) for F_5:7_ from Humai 15 and Mingxian 169 cross.

**Populations**	**Chr**	**Data set**	**Left marker**	**Right marker**	**LOD**	**PVE (%)**	**Add^b^**
F_2:3_	2B	rAUDPC2013_TS	Xbarc160	Xbarc167	7.5	19.7	−13.1
	2B	rAUDPC2013_YL	Xbarc160	Xbarc167	11.5	26.2	−13.9
	2B	rAUDPC2013 Ave	Xbarc160	Xbarc167	8.9	24.5	−11.9
	6A	rAUDPC2013_TS	Xwmc201	Xgwm570	3.4	8.3	−7.8
	6A	rAUDPC2013_YL	Xbarc113	Xgwm553	3.6	8.1	−5.5
	6A	rAUDPC2013_Ave	Xwmc201	Xgwm570	3.6	8.5	−6.2
F_5:7_ sub–population	2B	MDS2016_JY	IWB26631	IWB40714	6.6	35.3	−21.7
	2B	MDS2016_TS	IWB26631	IWB40714	6.5	34.5	−18.6
	2B	MDS2016_YL	IWB26631	IWB40714	10.6	37.6	−19.8
	2B	MDS2017_JY	IWB26631	IWB40714	5.1	27.8	−21.5
	2B	MDS2017_TS	IWB26631	IWB40714	8.1	52.2	−24.3
	2B	MDS2017_YL	IWB26631	IWB40714	8.8	45.8	−23.6
	2B	MDS_Ave	IWB26631	IWB40714	8.8	46.6	−22.5
	3B	MDS2016_JY	IWB66970	IWB23457	5.6	8.9	−11.2
	3B	MDS2016_YL	IWB66970	IWB23457	2.8	6.3	−8.1
	4B/6A	MDS2016_JY	IWB12138	IWB36089	2.7	6.3	−10.3
	4B/6A	MDS2016_YL	IWB6225	IWB7511	2.8	12.2	−10.2
	4B/6A	MDS2017_JY	IWB8350	IWB46525	3.4	27.4	−21.3
	4B/6A	MDS2017_TS	IWB13412	IWB8013	3.1	14.9	−12.9
	4B/6A	MDS2017_YL	IWB8350	IWB46525	2.8	12.2	−12.7
	7D	MDS2016_JY	IWB69295	IWB17584	4.7	23.7	17.7
	7D	MDS2016_TS	IWB69295	IWB17584	4.6	22.3	14.9
	7D	MDS2016_YL	IWB69295	IWB17584	7.7	23.3	15.6
	7D	MDS2017_YL	IWB69295	IWB17584	3.4	13.4	12.7
	7D	MDS_Ave	IWB69295	IWB17584	4.1	16.9	13.6
F_5:7_ RILs	2B	MDS2016_JY	IWB34732	IWA1981	25.2	47.2	−23.0
	2B	MDS2016_TS	IWB34732	IWA1981	16.3	34.2	−17.2
	2B	MDS2016_YL	IWB34732	IWA1981	18.3	37.6	−17.7
	2B	MDS2017_JY	IWB34732	IWA1981	17.7	36.5	−21.7
	2B	MDS2017_TS	IWB34732	IWA1981	23.5	45.8	−25.2
	2B	MDS2017_YL	IWB34732	IWA1981	19.5	39.6	−22.7
	2B	MDS_Ave	IWB34732	IWA1981	23.1	44.7	−21.3
	3B	MDS2016_TS	Xbarc87	IWB39254	3.1	7.8	−8.0
	3B	MDS2017_JY	Xbarc87	IWB39254	3.9	9.7	−10.9
	3B	MDS2017_TS	Xbarc87	IWB39254	4.4	10.8	−12.0
	3B	MDS2017_YL	Xbarc87	IWB39254	3.8	9.6	−10.8
	3B	MDS_Ave	Xbarc87	IWB39254	3.3	8.3	−8.9
	4B	MDS2016_JY	AX-111150955	IWB61846	2.6	6.7	−8.6
	4B	MDS2016_TS	IWB61846	Xgwm251	3.2	8.0	−8.4
	4B	MDS2017_JY	AX-111150955	IWB61846	2.7	6.7	−9.4
	4B	MDS2017_TS	IWB61846	Xgwm251	2.9	7.3	−10.2
	4B	MDS2017_YL	IWB61846	Xgwm251	3.1	7.6	−10.1
	4B	MDS_Ave	IWB61846	Xgwm251	2.9	7.4	−8.6

*^a^YL, TS, and JY denote Yangling, Tianshui and Jiangyou, respectively; 2013, 2016, and 2017 represent the populations grown during 2012-2013, 2015-2016 and 2016-2017 cropping season*.

*bLOD, logarithm of odds score*.

*cPVE, percentage of the phenotypic variance explained by individual QTL*.

*dAdd^b^, additive effect of resistance allele, where a negative value indicates that the allele was inherited from Humai 15, and a positive value indicates that the allele was from Mingxian 169*.

### Linkage maps and QTL analysis of sub-population

To reduce the cost, 45 RILs were randomly selected and genotyped with a 90K SNP array, where 9914 of 81,587 SNPs loci exhibited polymorphism between the parents, with severe segregation distortion (*P* < 0.001) in the 45 RILs. Among the polymorphic SNPs, 9361 SNPs were retained after removing more than 10% of the missing data for the sub-population. We found that these SNPs were assigned to 1405 bins because 7956 SNPs were redundant. Finally, 1339 SNPs (66 unlinked SNPs were removed) were distributed unevenly among 39 linkage groups, which spanned a total length of 4418.2 cM and they covered all 21 chromosomes.

Four QTL loci were identified by analyzing genotypic data based on the 90K SNP arrays for four chromosomes comprising 2BC, 3BS, 4B/6A, and 7DS, i.e., *QYrhm.nwafu-2BC, QYrhm.nwafu-3BS, QYrhm.nwafu-4B/6A*, and *QYrmx.nwafu-7DS*, respectively (Table 2, Figure 2). The resistance alleles for the QTLs on 2BC, 3BS, and 4B/6A were contributed by Humai 15, whereas *QYrmx.nwafu-7DS* came from Mingxian 169. A major and consistent QTL for stripe rust resistance, i.e., *QYrhm.nwafu-2BC*, was flanked by *IWB26631* and *IWB40714* with genetic distances of 2.5 and 1.5 cM, where it explained 27.8–52.2% of the phenotypic variation across all environments.

### KASP marker conversion and QTL analysis of the population of rils

Based on the map and QTL for the sub-population, the SNPs on chromosomes 2B, 3B, and 4B/6A converted into KASP markers and SSR markers were screened in the parents and 20 RILs in order to confirm their polymorphisms. Next, 13 SSR and 30 KASP stable polymorphism markers were selected to screen all 177 RILs, and three linkage groups were constructed based on the LOD of 2.5 set in the permutation test (Table 2, Figure 2). The majority of the phenotypic variation in this mapped population was explained by *QYrhm.nwafu-2BC*, which was localized on chromosome arm 2BC, where the QTL was flanked by the markers *IWB34732* and *IWA1981*, and it explained 36.5–47.2% of the phenotypic variation across all environments. On the short arm of chromosome 3B, *QYrhm.nwafu-3BS* was located in a 2.0 cM interval spanned by the SNP markers *Xbarc87* and *IWB39254*, where it explained 7.8–10.8% of the phenotypic variation in four environments. The third QTL, *QYrhm.nwafu-4B*, had a minor effect on MDS in all environments, except in Yangling2016, and it was localized in an interval of 3.4 cM on chromosome 4BL, where it was flanked by SNP markers *AX-111150955* and *Xgwm251*, and it explained 6.7 to 8.0% of the phenotypic variation with respect to stripe rust resistance.

### Candidate genes in *QYrhm.nwafu-2BC* and their contributions to apr in stripe rust

The interval of *QYrhm.nwafu-2BC* contained 46 genes (24 high confidence genes and 22 low confidence genes) based on iwgsc_refseqv1.0 annotations. Among these genes, 28 had homologs with annotated genes in TGAC. The TPM values related to biotic stress (stripe rust, *Fusarium* head blight, powdery mildew, and *Septoria tritici*) and abiotic stress (phosphorus starvation, drought, and heat) were retrieved. Eight genes out of them with a maximum value under 0.5 TPM (Borrill et al., 2017) were regarded as unexpressed genes and filtered out. The remaining 20 genes are shown in Figure S1. Based on the SNP markers in the interval of *QYrhm.nwafu-2BC*, two genes were identified in this region. According to the gene description and TPM value with respect to stripe rust stress, *TraesCS2B01G223600* and *TraesCS2B01G225000* encoding a protein kinase and a disease resistance protein with an ATP-binding site, respectively, were identified as candidate genes that might be involved with stripe rust resistance (Table 3).

**Table 3:** Candidate genes corresponding to *Qyrhm.nwafu-2BC* on chromosome 2B from 212579362 to 215204341 bp.

**Marker**	**gene-ID**	**Human-readable description**
AX-109861696	TraesCS2B01G222900	Receptor-like protein kinase
	TraesCS2B01G223000	Zinc finger FYVE domain-containing protein 1
	TraesCS2B01G223100	Transcription factor jumonji (jmjC) domain-containing protein
AX-11634843, AX-109974220	TraesCS2B01G223200	Adenylyl-sulfate kinase
	TraesCS2B01G223300	NHL domain protein
	TraesCS2B01G223400	40S ribosomal protein S13
	TraesCS2B01G223500	nuclear factor Y, subunit B6
AX-94645586, AX-94542446, AX-108923155	TraesCS2B01G223600	Protein kinase domain
AX-94631148	TraesCS2B01G223700	GDP-fucose protein O-fucosyltransferase 2
	TraesCS2B01G306300LC	Phosphoglycerate kinase
AX-111497698	TraesCS2B01G223900	DUF868 family protein (DUF868)
	TraesCS2B01G306700LC	26S proteasome non-ATPase regulatory subunit 3
AX-94916490	TraesCS2B01G224000	Phenylalanine ammonia-lyase
AX-89682408, AX-111710157	TraesCS2B01G224100	Pentatricopeptide repeat-containing protein
	TraesCS2B01G224300	Phenylalanine ammonia-lyase
	TraesCS2B01G224400	Ubiquitin thioesterase
	TraesCS2B01G306800LC	Retrovirus-related Pol polyprotein from transposon TNT 1-94
	TraesCS2B01G224500	WD-repeat protein, putative
AX-111678893	TraesCS2B01G224600	Pre-mRNA-splicing factor 18
	TraesCS2B01G224700	YABBY transcription factor
AX-94420966	TraesCS2B01G224800	Transporter-related family protein
AX-108821454, AX-95631734	TraesCS2B01G224900	Arginine/serine-rich coiled-coil 2
AX-112285965	TraesCS2B01G225000	Disease resistance protein
	TraesCS2B01G306900LC	pre-mRNA-splicing factor
	TraesCS2B01G307000LC	Transposon protein, CACTA, En/Spm sub-class
	TraesCS2B01G307200LC	NAC domain-containing protein, putative
AX-110096955	TraesCS2B01G225100	Vacuole membrane-like protein
AX-94992854	TraesCS2B01G225200	Protein TIME FOR COFFEE

### Haplotype analysis in the interval of *QYrhm.nwafu-2BC*

**Figure 3:**
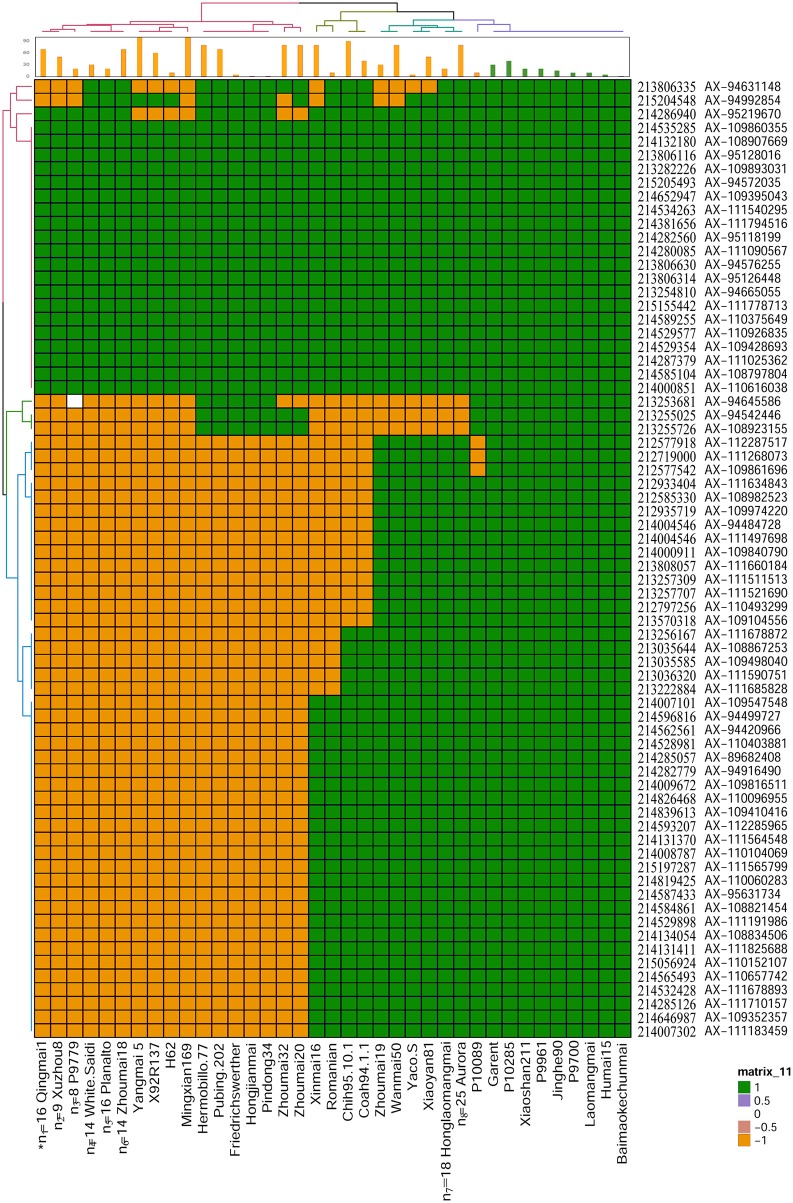
Haplotypes of 149 common wheat cultivars in the *QYrhm.nwafu-2BC* interval. Two-dimensional (row and column) hierarchical clustering analysis was performed to group lines into haplotypes, as indicated by the colorized dendrogram along the top X-axis and the similarly marker order is shown along the left Y-axis. The matrix comprises 660K genotypic data where SNPs with the same alleles as Humai 15 allele are shaded in green, whereas SNPs with different alleles to Mingxian 169 are shaded in orange. The name and physical position of each 660K probe were anchored to the physical position based on IWGSC_refseqv1.0. The mean stripe rust disease severity score for each line is shown in the bar chart along the top X-axis. *n, Numbers of lines collapsed into a single haplotype (Table S2).

Based on the flanking markers *IWB34732* and *IWA1981* (212.58–215.20 Mb) in the interval of *QYrhm.nwafu-2BC*, 70 probes in the 660K SNP array were located in this region. Four different haplotype groups were clustered in 149 wheat varieties or lines (Figure 3). Several stripe rust resistance derivatives, including Baimaokechunmai, Jinghe 90, Garent, and P10285, were found to cluster with the haplotype of Humai 15. We found that 48/149 varieties had the same haplotype as Humai 15, except in the interval of 212.57–215.20 Mb. The haplotype of Mingxian 169 was unique among the 149 wheat lines. In addition, a quarter of the wheat cultivars or lines contained the allele from Mingxian 169 and they shared less alleles with Humai 15. The wheat lines with the haplotype of Humai 15 had high or intermediate resistance to stripe rust. The remaining wheat lines were highly susceptible, with the exceptions of H62, Friedrichswerther, Pindong 34, and Yaco “S”.

### Confirmation of chromosome structure variation by FISH analysis

**Figure 4:**
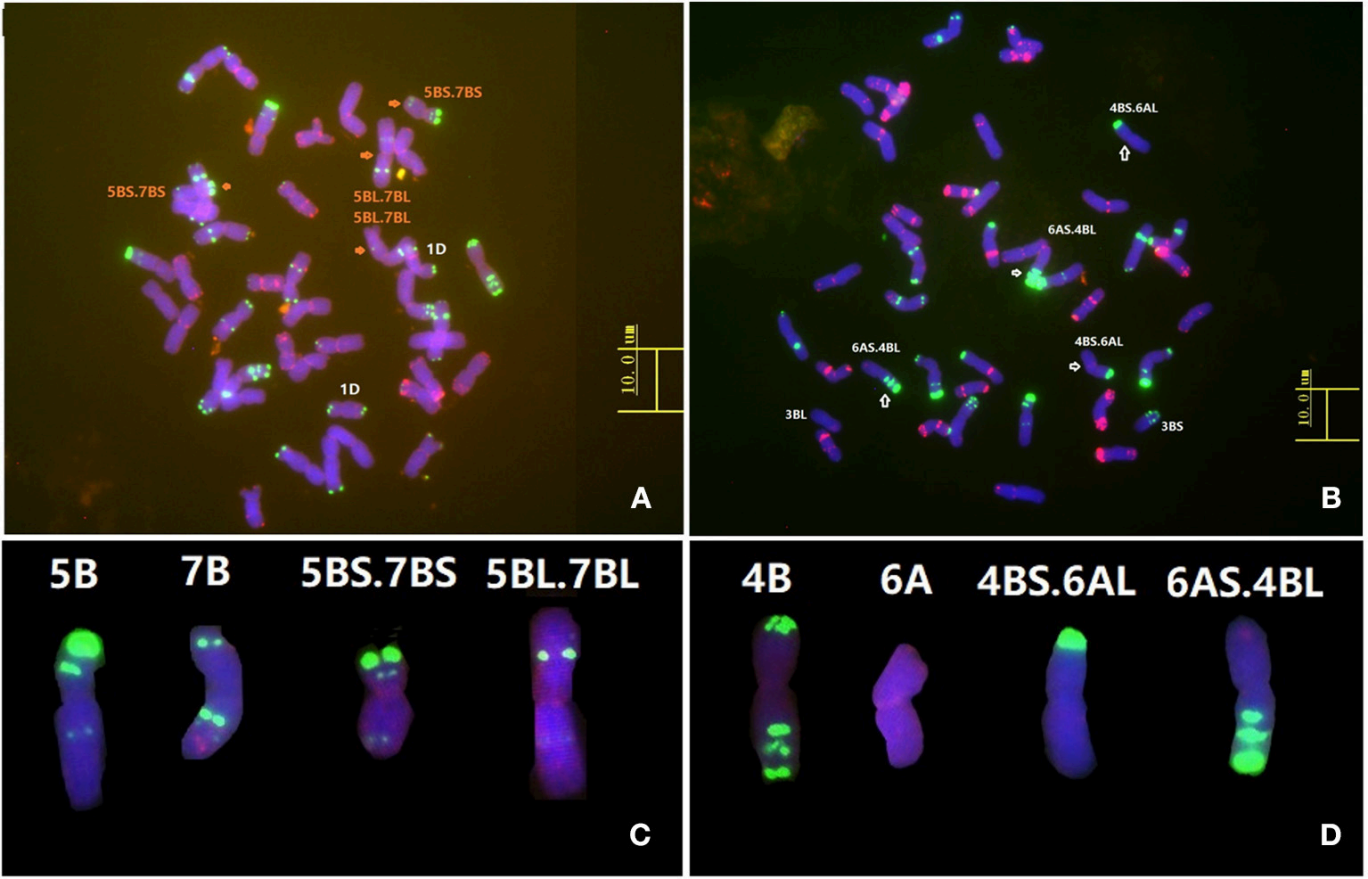
Fluorescence *in situ* hybridization (FISH) analysis of wheat lines Humai 15 **(A)** and Mingxian 169 **(B)**. Karyotypes of all the wild type chromosomes and translocations of 5B **(C)**, 7B **(C)**, 4B **(D)**, and 6A **(D)**. FISH was conducted using Oligo-pTa535 (*red*) and Oligo-pSc119.2 (*green*) as probes.

FISH analysis using the resistant parent Humai 15 confirmed the presence of a T5B-7B reciprocal translocation (Figure 4A). FISH analysis of Mingxian 169 confirmed the T4B-6A reciprocal translocation (Figure 4B). The translocation was compared with probes in the root tip metaphase chromosomes of Chinese Spring (Figures 4C,D). This result agreed with the genetic linkage map constructed with the SSR markers and 90K SNP array.

## Discussion

Previous studies have demonstrated that Humai 15 exhibited typical APR to stripe rust according to seedling and field tests since 2008. Mingxian 169 was highly susceptible in all stages and the *Yr18* allele was identified by detecting *cssfr3* and *cssfr5* (Han et al., 2012; Zeng et al., 2012). In the present study, the resistance to stripe rust in Humai 15 was characterized as quantitative inheritance and we identified three QTLs based on phenotypic data and genotyping of the population of RILs. The use of high density SNP array screening for a whole population is the most efficient way of mapping QTLs but it is highly expensive. Thus, in order to reduce the cost, we used a small population for screening with a 90K SNP array as described by Wang (2014), where a population of 40 RILs was employed to verify QTLs with PVEs of 20–30% on specific chromosomes based on the marker density at 5 cM. In the present study, 45 RILs were randomly selected and genotyped using a 90K SNP array and four QTLs were mapped onto chromosomes 2B, 3B, 4B/6A, and 7D. In addition, bulked segregant analysis with a 660K SNP array detected QTLs on chromosomes 2B, 4B, and 6A, with minor QTLs on chromosomes 3B and 7D (Figure S2). The flank linked markers were then screened in all of the RILs on chromosomes 2B, 3B, and 4B/6A. The results were consistent, thereby demonstrating that the rapid detection of QTL based on a small population is a viable and stable method.

*QYrhm.nwafu-2BC* explained 34.2–47.2% of the phenotypic variation, where it was flanked by the KASP markers *IWB34732* and *IWA1981. QYrhm.nwafu-2BC* was located on the centromere of chromosome 2B, which is a resistance gene rich-region. According to a previous genome-wide association study (Maccaferri et al., 2015), *Yr41* and *YrF* were detected on chromosome 2BS with a confidence interval of 29.6–47.7%. *Yr41* confers all-stage resistance but it is inefficient for the predominant Chinese *Pst* races (Zeng et al., 2015). *YrF* is a gene associated with all-stage resistance and APR, where it is designated as *QYr.cim-2BS* in Francolin#1, and it confers moderate resistance in Mexico and Sichuan province, China. *QYr.caas-2BS* was derived from Pingyuan50 as a minor QTL with a PVE of 5.1–9.5% for stripe rust in Gansu province, but not in Sichuan province (Lan et al., 2010). Chen et al. (2012) detected two major QTLs on chromosome arm 2BS in IDO444, where *QYrid.ui-2B.2* was flanked by molecular markers *Xgwm429* and *Xbarc91*, and PVE was 31%, but it was inconsistent in two environments. *QYr.tam-2BL* was detected on the long arm of chromosome 2B in TAM111, where it was flanked by *wPt6242* and *wPt6471*, and it had PVEs of 13–63% and 40.5% for stripe rust in adult plants and seedling plants, respectively. Mallard et al. (2005) also detected *QYr.inra-2B.1* on the centromere of chromosome 2B in Camp Remy and it explained PVEs of 42–61% in adult plants. According to the characteristics determined in the present study, as well as the PVEs and origin of the QTL, *QYrhm.nwafu-2BC* may be a novel QTL and more experiments should be conducted to confirm whether this is the case.

*QYrhm.nwafu-3BS*, a minor effect QTL, was located on chromosome arm 3BS and close to the SSR marker *Xbarc87*. Previous studies have reported many QTLs or genes (*Yr30, Yr57, Yrns-B1*, and *QYr.uga-3BS*) on chromosome 3BS, and several loci were shown to be *Yr30* in different wheat varieties (Singh et al., 2000; Suenaga et al., 2003; Yang et al., 2013; Basnet et al., 2014; Lan et al., 2014). The APR gene *Yr30/Sr2/Lr27* with a morphological marker for pseudo-black chaff was located in the terminal region of chromosome 3BS. *Yrns-B1* derived from Lgst.79-74 is a major APR gene and it is located close to the SSR marker *Xgwm493* (Börner et al., 2000). Humai 15 exhibits high resistance to stripe rust and it resists pseudo-black chaff at the adult plant stage. Due to its localization in a specific region and the PVEs of the genes, *QYrhm.nwafu-3BS* is different from *Yr30* and *Yrns-B1*.

*QYrhm.nwafu-4BL* was derived from Humai 15 and it accounted for 6.7 to 8.0% of the PVE to stripe rust, where it was flanked by *AX-111150955* and *Xgwm251*. In the same region, *Yr50* was reported to be associated with *Xbarc1096* and *Xwmc47*, and it was effective against CYR32 and CYR33 (Liu et al., 2013; Rosewarne et al., 2013; Maccaferri et al., 2015). Moreover, several major QTL genes (*Yr62* in PI 192252, *QYr.jic-4B* in Alcedo, and *QYrus.vt-4BL* in USG 3555) related to stripe rust at the adult plant stage have been reported (Jagger et al., 2011; Christopher et al., 2013; Lu et al., 2014). *QYr.sun-4B* is derived from the Australian wheat cultivar Janz and it exhibits minor variation (9.4–16.8%). It is flanked in the interval containing *wPt-8543, Xgwm368*, and *Xwmc238*, which covers the interval of *QYrhm.nwafu-4BL* (Zwart et al., 2010). *QPst.jic-4B* is derived from the UK winter wheat cultivar Guardian and it was mapped onto the region between *Xwmc652* and *Xwmc692* in the F_3_ population with a PVE of 12% (Melichar et al., 2008). Comparisons of the locations of the molecular markers indicate that *QYrhm.nwafu-4B, QYr.sun-4B*, and *QPst.jic-4B* are likely to be the same genes.

The QTL on the chromosome arm 7DS in Mingxian 169 was *Yr18* according to genotyping based on a 90K SNP array and identification of the gene-specific markers *cssfr3* and *cssfr5*.

According to the annotation of the Chinese Spring reference sequence (IWGSC refseq version 1.0 and TGAC V1), there are 28 genes in the interval of *QYrhm.nwafu-2BC*. Two cloned APR genes comprising *Yr18*/*Lr34* and*Yr36* are an ABC transporter and a protein kinase, respectively (Fu et al., 2009; Krattinger et al., 2009). The gene TraesCS2B01G223600, which encodes a protein kinase with protein kinase, ATP-binding serine/threonine-protein kinase, and protein phosphorylation activities, could be the candidate gene. In addition, TraesCS2B01G225000 encodes a disease resistance protein with an NB-ARC motif and ADP-binding domain. NB-ARC is a signaling motif related to cell death in plant R genes involved with cell death (Biezen et al., 1998; Takken et al., 2006). Traes_2BS_0EFFAAE28 and TraesCS2B01G225000 are likely to contribute to enhanced resistance and the hypersensitive response to stripe rust via the binding and hydrolysis of ATP to induce resistance signaling. TraesCS2B01G224000 and TraesCS2B01G224300 encode phenylalanine ammonia-lyases involved with the biosynthesis of polyphenol compounds such as flavonoids and lignin in plants in response to various stimuli such as tissue wounding, pathogenic attack, low temperature, and hormones (Huang et al., 2010). Thus, TraesCS2B01G224000 and TraesCS2B01G224300 are involved in the basal defense responses, which agreed with the results of transcriptome analyses based on responses to biotic and abiotic stresses (Figure S1).

Haplotype analysis to determine stripe rust resistance gene or loci in wheat cultivars can provide information regarding the frequency and reliability of wheat germplasm and varieties, as well as pedigrees (Yu et al., 2012). Several haplotype groups have been identified in wheat lines to screen the interval comprising the *QYrhm.nwafu-2B* region. These wheat cultivars shared the same haplotype as Humai 15 and they exhibited comparable resistance to stripe rust, so they may contain the QTL gene on chromosome 2B. Some wheat lines had different haplotypes at several alleles compared with Mingxian 169 but similar reactions to stripe rust. However, many of the wheat lines with these haplotypes exhibited high susceptibility to stripe rust, such as H62, 92R137, and Pindong 34, where in H62, 92R137, and Pindong 34, *YrH62* mapped onto chromosome 1B, *Yr26* onto chromosome 1BL, and *Yr61* onto chromosome 7A, respectively (Ma et al., 2001; Zhou et al., 2014; Wu et al., 2018). APR is controlled by several genes with complex mechanisms, and thus screening the entire genomic sequence is a better experimental approach for detecting variants or haplotypes to identify the positions of candidate genes (Johnson et al., 2001).

## Conclusion

Humai 15 is an early maturing variety of spring wheat developed in 2006 and it is suitable for growing at high altitudes in Qinghai province. Humai 15 is susceptible to stripe rust in both the field and greenhouses in the seedling stage, whereas it is highly resistant to stripe rust in the field in the adult stage. In the present study, we developed a population of RILs derived from Humai 15 and Mingxian 169 to detect QTLs that confer stripe rust resistance. We conducted 90K SNP array screening using a sub-population and identified three QTLs in Humai 15 and one QTL Mingxian 169, which were located on chromosomes 2B, 3B, and 4B/6A, and 7D, respectively. Based on a consistent genetic map, SSR and KASP markers were used to validate and saturate the QTLs in 177 F_6:7_ RILs. A major QTL gene *QYrhm.nwafu-2BC* was localized in the marker interval between *IWB34732* and *IWA1981*, where it explained 34.2–47.2% of the phenotypic variation. Other minor genes from Humai 15 were detected on chromosomes 3BS and 4BL, which were sensitive to the environment. One QTL gene from Mingxian 169 corresponding to *Yr18* was identified using the gene-specific markers cssfr3 and cssfr5. In addition, T4B-6A was detected in Mingxian 169 based on a genetic map of *QYrhm.nwafu-4BL*. The most frequent translocation, T5B-7B, was also detected in Humai 15 based on a genetic linkage map with SSR markers and a 90K SNP array. Translocations in Mingxian 169 and Humai 15 were confirmed by FISH.

## Author contributions

F-PY conducted the experiments, analyzed the data, and wrote the manuscript. F-PY, Q-DZ, and J-HW identified the resistant parental line, made the cross and participated in the field experiments. B-PL and J-HW participated in field experiments and contributed to the genotyping experiment. Q-LW and Q-DZ assisted with analyzing the data. Z-JY carried out the FISH analysis. Z-SK, X-HC, and D-JH conceived and directed the project and revised the manuscript.

### Conflict of interest statement

The authors declare that the research was conducted in the absence of any commercial or financial relationships that could be construed as a potential conflict of interest.

## Supplementary material

Figure S1 Heat map based on 20 genes in the interval of *Qyrhm.nwafu-2BC* on chromosome 2B under biotic and abiotic stresses.

Figure S2 Polymorphic SNPs between bulk samples distributed on 21 chromosomes using a 660K chip array and the proportion on one chromosome SNP in the bulk samples and parents using a 660K chip. Gray and white columns indicate the numbers of polymorphic SNPs in parents and bulk samples, respectively.

Table S1 Correlation coefficients (*r*) of the mean MDS in the six environments for the population of RILs derived from Mingxian 169 × Humai 15. ^*^All of the r values are significant at *P* < 0.001.

Table S2 Wheat lines collapsed into a single haplotype.
