# Supplementary figures and images for: QTL Mapping and Validation of Adult Plant Resistance to Stripe Rust in Chinese Wheat Landrace Humai 15
Source: Front Plant Sci. 2018 Jul 5;9:968. doi: 10.3389/fpls.2018.00968 (PMC6041984; doi:10.3389/fpls.2018.00968)

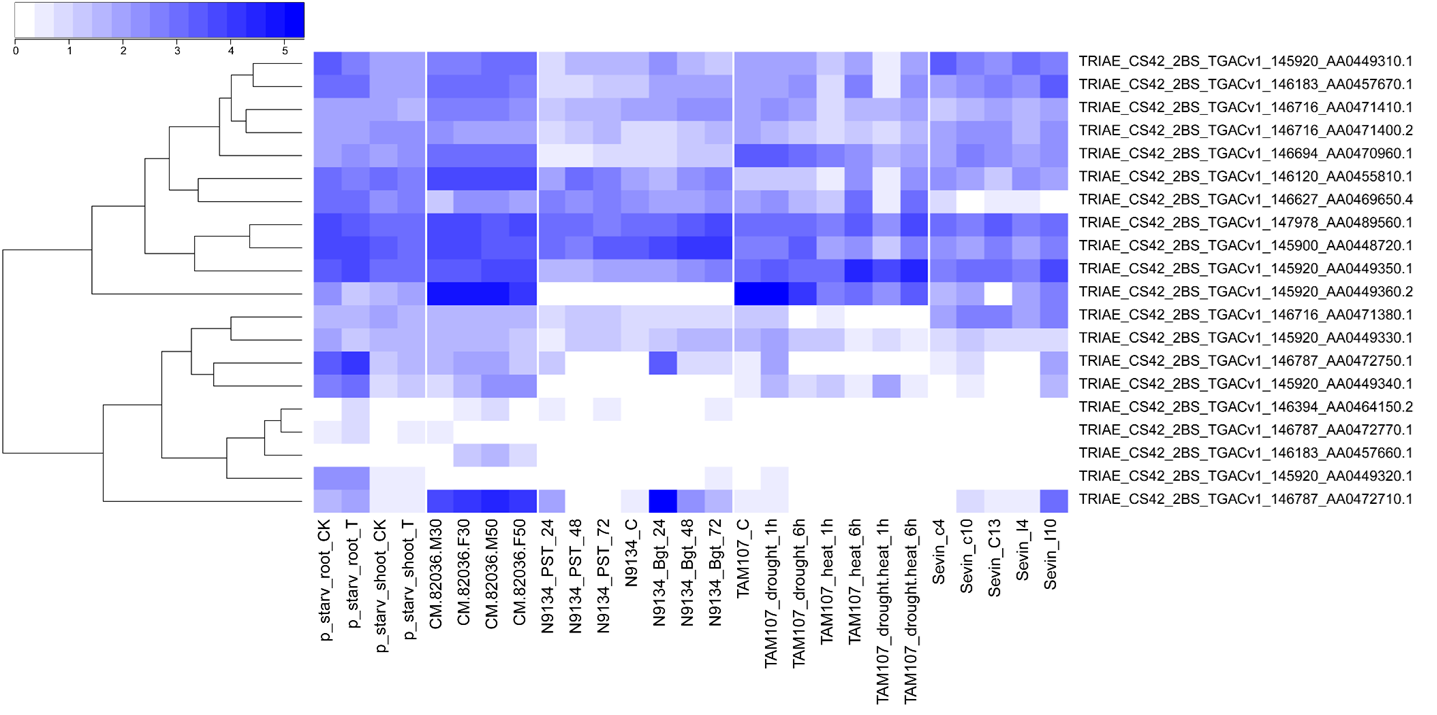

Supplement: Figure S1 — Heat map based on 20 genes in the interval of Qyrhm.nwafu-2BC on chromosome 2B under biotic and abiotic stresses. [file Image_1.TIF]

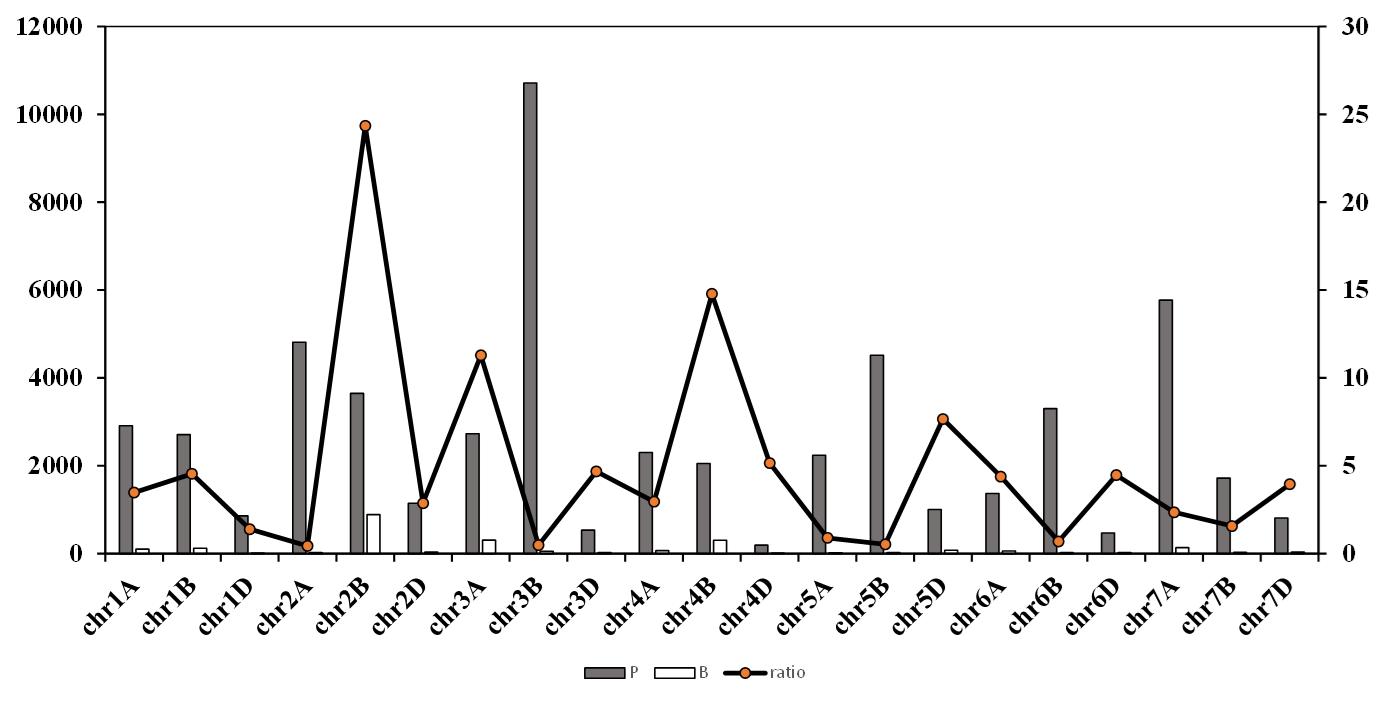

Supplement: Figure S2 — Polymorphic SNPs between bulk samples distributed on 21 chromosomes using a 660K chip array and the proportion on one chromosome SNP in the bulk samples and parents using a 660K chip. Gray and white columns indicate the numbers of polymorphic SNPs in parents and bulk samples, respectively. [file Image_2.TIF]
