# Supplementary material for: QTL Mapping and Validation of Adult Plant Resistance to Stripe Rust in Chinese Wheat Landrace Humai 15
Source: Front Plant Sci. 2018 Jul 5;9:968. doi: 10.3389/fpls.2018.00968 (PMC6041984; doi:10.3389/fpls.2018.00968)
Supplement: Table S1 — Correlation coefficients (r) of the mean MDS in the six environments for the population of RILs derived from Mingxian 169 × Humai 15. *All of the r values are significant at P < 0.001. [file Table_1.DOCX]

**Table S1.** Correlation coefficients (*r*) of mean MDS of the Mingxian 169 × Humai 15-derived RIL population in the six environments

| Environment (Location, year) | Jiangyou 2016 | Tianshui 2016 | Yangling 2016 | Jiangyou 2017 | Tianshui 2017 | Yangling 2017 |
| --- | --- | --- | --- | --- | --- | --- |
| Jiangyou 2016 | 1.00 |  |  |  |  |  |
| Tianshui 2016 | 0.90 | 1.00 |  |  |  |  |
| Yangling 2016 | 0.91 | 0.90 | 1.00 |  |  |  |
| Jiangyou 2017 | 0.90 | 0.91 | 0.87 | 1.00 |  |  |
| Tianshui 2017 | 0.84 | 0.89 | 0.81 | 0.92 | 1.00 |  |
| Yangling 2017 | 0.88 | 0.90 | 0.86 | 0.95 | 0.92 | 1.00 |

*All of the r values were significant at P < 0.001
