# Supplementary material for: QTL Mapping and Validation of Adult Plant Resistance to Stripe Rust in Chinese Wheat Landrace Humai 15
Source: Front Plant Sci. 2018 Jul 5;9:968. doi: 10.3389/fpls.2018.00968 (PMC6041984; doi:10.3389/fpls.2018.00968)
Supplement: Table S2 — Wheat lines collapsed into a single haplotype. [file Table_2.DOCX]

**Table S2** The wheat lines collapsed into a single haplotype

| **n_1_** | **Qinmai 1** | n_4_ | Extra Kolben Heinego | n_7_ | Jinan 16 |
| --- | --- | --- | --- | --- | --- |
| n_1_ | Shanhe 6 | n_4_ | Hindi | n_7_ | Iwa 8606392 |
| n_1_ | Yumai 15 | n_4_ | Felasit Caiek | n_7_ | Lumai 12 |
| n_1_ | Abyssinian 43 | n_4_ | Sunset | n_7_ | Kezhuang |
| n_1_ | Russia | n_4_ | Purplestraw | n_7_ | Huimaoafu |
| n_1_ | Grifone 235 | n_4_ | Trigo Blanco | n_7_ | Jingnong 79 13 |
| n_1_ | Marrocos 163608 | n_4_ | Qro94.1.1 | n_7_ | Early Red Fife |
| n_1_ | Syria 1740 | n_5_ | **Planalto** | n_7_ | John Brown |
| n_1_ | Xiaohongmai | n_5_ | Robadinho | n_7_ | Bolton |
| n_1_ | Qingchun 5 | n_5_ | Austral | n_7_ | Aimengniu 5 |
| n_1_ | Shannong 1 | n_5_ | Oax92.1.1 | n_7_ | Indiano Mocho 6296 |
| n_1_ | Xuzhou 25 | n_5_ | Red Bobs 222 | n_7_ | Ghabagheb |
| n_1_ | Shannong 9 | n_5_ | Kennedy Harlan J.R 38 | n_7_ | Red Kamsereti |
| n_1_ | Yumai 47 | n_5_ | Iragi | n_7_ | Txl92.1.1 |
| n_1_ | Zhoumai 13 | n_5_ | Egypto 2100 | n_7_ | Beladi 24 B |
| n_1_ | Zhengzhou 5 | n_5_ | Mich89.1.3 | n_8_ | **Aurora** |
| n_2_ | **Xuzhou 8** | n_5_ | Gluyas Early | n_8_ | Rooi Spitskop |
| n_2_ | Zhengzhou 17 | n_5_ | Roter Kolben Dinkel | n_8_ | Fengkang 7 |
| n_2_ | Youmanghong 7 | n_5_ | Alshvede | n_8_ | Dongfanghong 3 |
| n_2_ | Xindong 20 | n_5_ | Lagodekhis Grdzel Tavtava | n_8_ | Kehan 10 |
| n_2_ | Maoyingafu | n_5_ | Mex94.10.1 | n_8_ | Kefeng 6 |
| n_2_ | Yumai 24 | n_5_ | Red Fife | n_8_ | Jimai 23 |
| n_2_ | Yumai 16 | n_5_ | Gharflor 1611 | n_8_ | Jinan 8 |
| n_2_ | Senmarq | n_6_ | **Zhoumai 18** | n_8_ | Lumai 5 |
| n_2_ | Anolito Bronco | n_6_ | Penny | n_8_ | Lumai 3 |
| n_3_ | P9779 | n_6_ | 2799:Ae | n_8_ | Lumai 11 |
| n_3_ | P9746 | n_6_ | Universal Ⅱ | n_8_ | Longmai 33 |
| n_3_ | Villosum | n_6_ | Sarsabaz | n_8_ | Lianmai 2 |
| n_3_ | P9752 | n_6_ | Salti Naimya | n_8_ | Hezuo 4 |
| n_3_ | P9827 | n_6_ | Lohari Y91 92 No.123 | n_8_ | Keqiang |
| n_3_ | P9769 | n_6_ | Montezuma Club | n_8_ | Chuangmai 22 |
| n_3_ | P9778 | n_6_ | HongDuanMang | n_8_ | Baihuomai |
| n_3_ | P9771 | n_6_ | Pue94.1 | n_8_ | Mishragani |
| n_4_ | **White Saidi** | n_6_ | Hudeiba 164 | n_8_ | Kenjiu 10 |
| n_4_ | Tol93.1.2 | n_6_ | Mex92.1.1.1 | n_8_ | Longmai 26 |
| n_4_ | 2821:Ae | n_6_ | 2911 H558 43 1480 Div6712 | n_8_ | Baiyoubao |
| n_4_ | Pusa 80 5 C | n_6_ | Bainong 791 | n_8_ | Chuangmai 42 |
| n_4_ | Oax93.1.1.1 | n_7_ | **Honglaomangmai** | n_8_ | Een1 |
| n_4_ | Red Beard | n_7_ | Jimai 3 | n_8_ | Punjabi Sarvati |
| n_4_ | Koelz W 8a | n_7_ | Jinan 4 | n_8_ | Wgru89 9 |
